# A Case Report of Tranexamic Acid for the Treatment of Chronic Subdural Hematoma in an 86-Year-Old Patient

**DOI:** 10.7759/cureus.66846

**Published:** 2024-08-14

**Authors:** Shatha Gharaibeh, Abdallah Shurman, Abeer Gharaibeh, Roy Torcuator

**Affiliations:** 1 Center for Cognition and Neuroethics, University of Michigan-Flint, Flint, USA; 2 Clinical Research, Insight Research Institute, Flint, USA; 3 Clinical Research, Insight Institute of Neurosurgery and Neuroscience, Flint, USA; 4 Neurosurgery, Insight Institute of Neurosurgery and Neuroscience, Flint, USA; 5 Neurosurgery, Insight Surgical Hospital, Warren, USA

**Keywords:** txa, surgery-preventing strategies, csdh, chronic subdural hematoma, tranexamic acid

## Abstract

Fibrinolytic and coagulative hyperactivity is proven to have a role in liquefaction and progression of chronic subdural hematoma (CSDH). Tranexamic acid was one of the pharmaceutical options that was explored, as it inhibits the hyper-fibrinolytic activity and reduces the vascular permeability in CSDH, leading to a gradual resolution of the hematoma. In this case study, we present a case of using tranexamic acid for CSDH treatment in an 86-year-old patient with co-morbidities. The complete resolution of the hematoma following using tranexamic acid in this case with no history of recurrence in two years follow-up supports its efficacy in CSDH treatment and may be considered as one of the strategies that help prevent surgeries.

## Introduction

Chronic subdural hematoma (CSDH) is an encapsulated collection of blood and fluid on the surface of the brain [1,2]. It is often revealed three weeks or more after a head trauma, and it is bilateral in 20% of cases [1,2]. The incidence of CSDH in the general United States (US) population is 5-58 per 100,000 [2-6]. Since it increases with age, the overall incidence of CSDH is also likely to rise by 2050 [2]. Markwalder's grading scale and Glasgow coma scale system (MGS-GCS) are used to assess the severity and progression of patients with CSDH, considering both the severity of clinical symptoms and the consciousness state score [7].

The treatment approach for CSDH may vary, with options ranging from conservative methods to surgical interventions [2,8,9]. However, the treatment choice depends on factors such as the patient’s overall health, their symptoms, and the volume of the hematoma [3,10]. The conservative approach involves implementing a wait-and-scan policy, during which patients are regularly monitored for neurological deterioration and hematoma expansion through follow-up imaging [5]. Conservative measures are generally used for managing asymptomatic patients while symptomatic patients with cerebral compression and an acceptable surgical risk are treated surgically [11,12]. Although surgery is still considered a straightforward and safe procedure in neurological impairment cases, the recurrence rate is relatively high [12-14]. Moreover, surgery is often not recommended for elderly patients due to their pre-existing comorbidities. Thus, there is considerable importance placed on finding a safe and efficient conservative treatment for CSDH [10].

Tranexamic acid (TXA) has shown effectiveness in reducing bleeding in various hemorrhagic conditions [15-17]. The only Food and Drug Administration (FDA)-approved usage for TXA is for heavy menstrual bleeding and short-term prevention in patients with hemophilia [16]. However, the drug was shown to reduce rates of mortality in urgent surgery in patients with acute trauma by reducing perioperative bleeding and reducing the need for blood transfusions [15]. Additionally, the use of TXA in upper gastrointestinal bleeding was associated with decreasing many complications including the need for blood transfusion, re-bleeding, or urgent surgeries [16,17]. Many other conditions seem to have benefited from using TXA in reducing bleeding such as knee arthroplasty, transurethral prostatic surgery, placental abruption, postpartum hemorrhage, ocular hemorrhage, and oral surgeries [15-17].

The onset of CSDH with mass effect is typically initiated by a minor traumatic event that tears the dural border cell layer. This tearing allows cerebrospinal fluid (CSF) and blood to leak into the subdural space. This leakage sets off a cascade involving inflammation, impaired coagulation, fibrinolysis, and angiogenesis [18]. TXA is one of many drugs that have been tested in the conservative management of CSDH [3,8,10,19]. It is hypothesized that TXA simultaneously has antifibrinolytic and anti-inflammatory effects by inhibiting plasminogen activator and plasmin and reducing vascular permeability and leukocyte migration in the outer hematoma membrane [2,3,8]. These changes lead to microbleeds which help resolve CSDH.

## Case presentation

An 86-year-old Caucasian male patient was presented to the neurosurgery clinic in May 2022 due to a history of a falling injury and CSDH in the right parietal lobe. The patient was clinically asymptomatic with an intact neurological exam and his GCS score was 15 (MGS-GCS grade 0). The first computed tomography (CT) imaging showed acute subdural hemorrhage in the right frontal and temporal convexity, which was measuring 5 mm at that time. Cerebral cortical contusion on the anterior inferior aspect of the right frontal lobe was also suspected. There was no midline shift nor depressed skull fracture seen.

**Figure 1:**
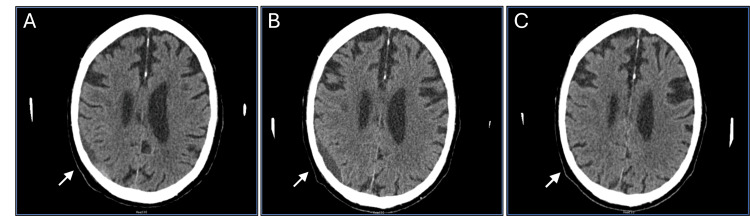
Follow-up CT scans show CSDH over time before and after TXA treatment (A & B) Follow-up CT scans show the progression in CSDH size and local mass effect over time before TXA treatment. (A) The size of the hematoma was 7 mm in June 2022, which indicates an increase in volume compared to the initial presentation. (B) The size of the hematoma was 11 mm in July 2022, which indicates an increase in the hematoma volume compared to the previous follow-up visit. (C) The CT scan shows complete resolution of the hematoma after the TXA treatment for one month. The previously noted subdural fluid collection in the right parietal area has completely resolved and the brain expanded in that area.

The follow-up CT scan showed interval progression in the size of the hematoma on the right parietal lobe (Figures 1A, 1B). The lesion appears to be more chronic in character with minimal local mass effect. The patient was clinically asymptomatic with an intact neurological exam and his GCS score was 15.

Considering that the patient had a progressing CSDH but is clinically asymptomatic with an intact neurological exam, and a history of co-morbidities, no neurosurgical intervention was recommended. The patient also reported that he doesn’t take any anticoagulant medication. Therefore, using TXA to treat CSDH was discussed with the patient after consultation with the cardiologist. The patient agreed with the plan and a TXA 650 mg tab once a day for a month was prescribed.

A new CT scan was ordered in September 2022 to compare pre-treatment and post-treatment using TXA for a month as shown in Figure 1C. There was interval resolution of the previously seen hypoattenuating extra-axial fluid collection overlying the right parietal lobe. There was no acute intracranial hemorrhage identified. The previously noted subdural fluid collection in the right parietal area has completely resolved and the brain was expanded in that area. The patient was clinically stable with no new neurologic findings on the exam and no need for neurosurgical intervention after two years of follow-up.

Past medical history

The patient is a former smoker who had a history of benign prostatic hypertrophy, type 2 diabetes mellitus, morbid obesity, essential hypertension, hard of hearing, macular degeneration, pure hypercholesterolemia, osteoarthritis with a history of bilateral knee and hip replacement, and coronary artery disease with a history of triple bypass surgery.

## Discussion

In this case report, an 86-year-old patient was treated with TXA with a dose of 650 mg tab once daily for a month, which resulted in complete resolution of his CSDH and no recurrence after two years of follow-up. The conservative management of CSDH is debatable [19]. There is no consensus on the optimal treatment or standard guidelines for deciding on a medication [4]. In patients with only moderate symptoms, treatment with TXA has been suggested [4]. Given the patient's age of 86 years, which is considered a morbidity factor in addition to the other comorbidities, conservative treatment was considered over surgical options. In this case study, we have shown that there was a complete CSDH resolution after TXA treatment with no history of recurrence in two years of follow-up. TXA has been used for CSDH previously and case studies were published at doses ranging from 650 mg to 1000 mg per day for one to three months [4,5,20].

In previous literature, seven patients were treated with TXA with six of them having no need for surgical interventions within 12 weeks after TXA treatment [4]. In another study, 20 cases of primary CSDH and 7 cases of recurrent CSDH following surgery were given TXA [19]. None of these patients had a complication or recurrence. Moscote-Salazar and colleagues reported a CSDH case of a 60‑year‑old patient who was managed conservatively by primary TXA achieving complete resolution [21]. In a prospective study of 27 patients, TXA was shown to be effective in the treatment of CSDH without the need for surgery [19]. There was only one patient in this study older than 80 years. In another study of 21 patients, the use of TXA reduced the mean volume of CSDH from 58.5 ml to 3.7 ml [8]. Another case of a 90-year-old female with a subdural hematoma measuring 130 mL with a mass effect and a midline shift of 7 mm was treated by the administration of TXA. After the full completion of a TXA course, the patient achieved baseline mobility. The final measurements revealed a final hematoma volume of 10 mL and a midline shift of less than 2 mm [22].

TXA was also used as a treatment after surgical interventions to reduce the recurrence rate [8]. In a published study, complete resolution was observed after TXA administration to three patients with recurrent CSDH following surgical intervention [23]. In another study, the residual volume of CSDH was reduced by 91.31% during oral TXA treatment for 14 patients who underwent surgeries primarily [24]. No patients developed delayed recurrence or expansion of their hematomas. Mikkelsen and colleagues reported a case of recurrent CSDH necessitating surgical intervention five times. Interestingly, intravenous TXA was administered after the fifth surgery, resulting in the resolution of both the symptoms and the hematoma [25].

On the contrary, a previous study showed no benefits of using TXA with the potential of increasing complication odds such as pulmonary thromboembolism and surgical site infection [26]. Another study showed no benefits in reducing the recurrence rate after burr hole surgery [27]. The variable results could be due to many factors that need to be studied further, including disease and patient characteristics. Nevertheless, it seems reasonable based on theoretical and clinical data that a subset of patients will benefit from TXA.

There are several indications for the use of drugs to promote hematoma absorption, including stable vital signs, MGS-GCS grade 0-2, less than 1 cm shift in the midline, multiple organ failure or coagulation dysfunction, and recurrence after surgery [13]. On the contrary, among the contraindications are MGS-GCS grade 3-4, severe compression of the brain and a shift over 1 centimeter in the midline, signs of brain herniation and consciousness disorders, and allergy or contraindication to the drug [13]. By applying these guidelines, the patient in this case study was a good candidate for using TXA, as his vital signs were stable, he was asymptomatic, and his GCS was 15. There was no midline shift nor depressed skull fracture seen, and he had no known drug allergies.

The pathophysiology of the CSDH is not yet fully understood, but current findings provide some insights. The subdural space comprises dural border cells, which have less tight junctions compared to the rest of the dura and arachnoid mater. Minor trauma initiates a cascade of events, starting with the cleavage of the dural border cell layer, leading to the CSF leakage into the subdural space. Injured cells release cytokines, which trigger inflammation that increases fibroblast activity and vascular endothelial growth factor (VEGF) production, resulting in chemokine release and immature capillary formation. This process allows extravasation of vascular contents into the newly formed hematoma cavity. Additionally, inflammatory mediators release tissue plasminogen activator (tPA), which leads to continuous bleeding and hyperfibrinolysis that is marked by increased fibrinogen degradation products and decreased plasminogen, leading to impaired platelet function and defective clotting [18]. TXA can potentially inhibit the fibrinolytic and inflammatory (kinin-kallikrein) systems simultaneously. By inhibiting the growth cascade of the hematoma and reducing its size at the same time, TXA may lead to CSDH resolution [8,28].

A deeper understanding of the underlying pathophysiological mechanisms is thought to assist in specifically targeting the dysregulated elements of coagulation cascades, which are believed to contribute to the growth of CSDH [20]. Further studies are necessary to include patients treated with TXA as a first-line treatment to prevent surgery or as a post-surgical treatment to prevent recurrent CSDH [5].

## Conclusions

Putting it all together, TXA may prevent the early stages of CSDH that can occur after head trauma. Therefore, it could be considered as primary medical treatment in patients with CSDH or following surgical treatment to prevent recurrence based on the described results in the previously published literature. TXA could be a valid conservative treatment in a small patient series, but for patients treated with antithrombotic or anticoagulant medication, the risk of increasing thromboembolic events is still to be evaluated. Clinical trials that can look at the efficacy of using TXA in CSDH treatment are still needed to provide evidence on the efficacy of TXA and the treatment guidelines.
